# Binding of the extracellular matrix laminin-1 to *Clostridioides difficile* strains

**DOI:** 10.1590/0074-02760220035

**Published:** 2022-06-17

**Authors:** Mayara Gil de Castro Santos, Camilla Nunes dos Reis Trindade, Rossiane Cláudia Vommaro, Regina Maria Calvalcanti Pilotto Domingues, Eliane de Oliveira Ferreira

**Affiliations:** 1 Universidade Federal do Rio de Janeiro, Departamento de Microbiologia Médica, Laboratório de Biologia de Anaeróbios, Rio de Janeiro, RJ, Brasil; 2 Universidade Federal do Rio de Janeiro, Instituto de Biofísica Carlos Chagas Filho e Instituto Nacional de Ciência e Tecnologia em Biologia Estrutural e Bioimagens, Laboratório de Ultraestrutura Celular Hertha Meyer, Rio de Janeiro, RJ, Brasil

**Keywords:** Clostridioides difficile, adhesion, laminin-1, virulence

## Abstract

**BACKGROUND:**

*Clostridioides difficile* is the most common cause of nosocomial diarrhea associated with antibiotic use. The disease’s symptoms are caused by enterotoxins, but other surface adhesion factors also play a role in the pathogenesis*.* These adhesins will bind to components of extracellular matrix.

**OBJECTIVE:**

There is a lack of knowledge on MSCRAMM, this work set-out to determine the adhesive properties of several *C. difficile* ribotypes (027, 133, 135, 014, 012) towards laminin-1 (LMN-1).

**METHODS:**

A binding experiment revealed that different ribotypes have distinct adhesion capabilities. To identify this adhesin, an affinity chromatography column containing LMN-1 was prepared and total protein extracts were analysed using mass spectrometry.

**FINDINGS:**

Strains from ribotypes 012 and 027 had the best adhesion when incubated with glucose supplementations (0.2%, 0.5%, and 1%), while RT135 had a poor adherence. The criteria were not met by RT014 and RT133. In the absence of glucose, there was no adhesion for any ribotype, implying that glucose is required and plays a significant role in adhesion.

**MAIN CONCLUSIONS:**

These findings show that in the presence of glucose, each *C. difficile* ribotype interacts differently with LMN-1, and the adhesin responsible for recognition could be SlpA protein.

*Clostridioides difficile* is an anaerobic spore-forming bacteria that is the most common cause of nosocomial antibiotic-associated diarrhea and pseudomembranous colitis, an acute gut inflammation.^1^ *C. difficile* infection (CDI) most commonly affects elderly patients with comorbidities, people taking broad-spectrum antibiotics and have been hospitalised for an extended period of time, and immunocompromised patients.^2^

The CDI begins with the acquisition of resistant spores, which shall occur in hospital and community, settings and initiate the germination process when they encounter a disrupted intestinal microbiota. *C. difficile* spores germinate in the gut once the dysbiosis is established, and several virulence factors are activated. The most common toxins that cause CDI are enterotoxin A (TcdA), cytotoxin B (TcdB) and binary toxin (CDT), although the CDT is only found in epidemic strains, such as the ribotype 027. They are encoded in the chromosomal pathogenicity locus (PaLoc)^1^ ^,^ ^3^ of the species’ and are efficiently produced in response to a variety of environmental cues after colonisation during the late log and stationary growth phases.^4^ Toxins cause inflammatory responses such as cell junction disruption, cytokine and mediator production, neutrophil infiltration, fluid release, actin filament rearrangement and cell death.^4^

The adhesion process is aided by various surface components on *C. difficile* that contribute to the colonisation of the intestine. Only a few of these structures have been described so far, including flagella components,^5^ cell wall proteins - CWP^6^ and the S-layer (SLPs) proteins, which are formed by the low molecular weight protein (LMW-SLP) and the high molecular weight protein (HMW-SLP).^6^ ^,^ ^7^

Several bacterial surface components, such as, fimbriae, outer membrane proteins (PME), capsular polysaccharides, lipopolysaccharides (LPS), pili and biofilm^8^ ^,^ ^9^ ^,^ ^10^ ^,^ ^11^ can be involved in host colonisation. Microbial surface components recognising adhesive matrix molecules (MSCRAMMs), which are surface proteins responsible for recognising the extracellular matrix (ECM) in the host, can also contribute for the bacterial virulence.^12^ ^,^ ^13^ These surface proteins can bind collagens, fibronectins and laminins, as well as provide structural and biochemical support to neighboring cells and regulate adhesion, migration, proliferation and differentiation.^13^ ^,^ ^14^ Laminin (LMN) is a glycoprotein with a molecular weight ranging from 400 to 900 kDa that is the primary component of the basal membrane (BM) that separates epithelial cells from the underlying stromal cells.^15^ ^,^ ^16^ This molecule is made up of three polypeptide chains (alpha, beta and gamma) that are bound together by disulfide bonds with an asymmetric arrangement.^16^ ^,^ ^17^ The new nomenclature has already discovered 19 distinct LMN isoforms - five alpha, four beta and three gamma variants - and some pathogens may use them for epithelial adherence and invasion, as well as evasion of the host immune response.^18^ LMN is composed by three short N-terminal arms and one long C-terminal arm. On the their C-terminus, all LMNs have five globular domains that are known to interact with microorganisms.^17^ Because LMN is found in the BM, it may be exposed to *C. difficile* during CDI, when toxins are released, disrupting or disorganising the epithelium, allowing the bacterium to colonise and persist in the colon. Bacteria must be able to recognise ECM during the pathogenic process.

As previously stated, several adhesins associated with *C. difficile* are involved in gut colonisation, however information on the adhesins involved in ECM recognition is limited. Fbp68, a fibronectin-binding protein, has been identified as a *C. difficile* colonisation factor that binds to fibrinogen, vitronectin, and manganese (Mn) in a Mn-dependent manner.^19^ ^,^ ^20^ CbpA, a collagen-binding protein found on the surface of *C. difficile*, was discovered. Recombinant CbpA has a high affinity for type I and V collagens with high affinity and has adhesive properties in gastrointestinal tissues.^21^ Another study found that *C. difficile* secretes Zmp1, a zinc-dependent metalloprotease that degrades fibronectin and fibrinogen in vitro in a zinc-dependent manner, cleaving two putative adhesins, CD2831 and CD3246.^22^ ^)^

MSCRAMMs are already well characterised in some bacterial pathogens and play an important role in the infectious process (adhesion, invasion, and evasion). However, in the case of *C. difficile*, the characterisation of these adhesins and their involvement during CDI remain unknown, as well as possible recognition by laminin-1 (LMN-1). The laminin receptor is highly conserved across eukaryotes and is involved in more than just cell-pathogen interactions. As a result, the goal of this study was to determine the ability of several *C. difficile* ribotypes found worldwide (012, 027 and 014), as well as two autochthonous isolates from Brazil (133 and 135) to adhere to LMN-1 and characterise the molecule responsible for this recognition.

## MATERIALS AND METHODS

*Bacterial strains and culture conditions* - All *C. difficile* isolates used in this study are from the Anaerobe Biology Laboratory’s Culture Collection (Instituto de Microbiologia Paulo de Góes, Universidade Federal do Rio de Janeiro, Brazil - UFRJ) (Table). Ribotypes 135 (SJ1 strain) and RT133 (HU09 strain) have been isolated from CDI patients in Brazil. The epidemic strains RT027 (R20291 strain; CDC ANA #2004016) and RT014 (1598) were included for comparison, as was the RT012 (CD630) strain. All strains were grown overnight at 37ºC in Brain Heart Infusion Broth - Pre- Reduced Anaerobic Sterilised (BHI-PRAS - 0.4 g/L cysteine, 10 µg/mL hemin (5.0 µg/mL), 4 mL/L resazurin and 10 µg/mL menadione; Oxoid^®^), in an anaerobic (80% nitrogen, 10% hydrogen, 10% carbon dioxide) cabinet (Coy Laboratory Products, INC.). In the formula of the BHI contains 2g/L of glucose (0.2%).

**TABLE:** *Clostridioides difficile* strains used in this study

Strains	Ribotypes	Toxigenic profile^*b*^	Clinical conditions	References
SJ1	135^*a*^	*tcd*A^*+*^ */tcd*B^*+*^ */*CDT^-^	SH, DM, hemorrhagic EVA	^23^
HU09	133^*a*^	*tcd*A^*+*^ */tcd*B^*+*^ */*CDT^-^	Crohn’s disease	^24^
1598	014	*tcd*A^*+*^ */tcd*B^*+*^ */*CDT^-^	HIV^+^	^25^
BI/NAP1^*c*^	027	*tcd*A^*+*^ */tcd*B^*+*^ /CDT^+^	-	^26^
630	012	*tcd*A^*+*^ */tcd*B^*+*^ */*CDT^-^	Pseudomembranous colitis	^27^

*a*: ribotypes of *C. difficile* exclusive from Brazil; *b*: polymerase chain reaction (PCR) was used to the detection of the toxin genes; *c*: BI/NAP1/027 strain (ANA #2004016) was kindly provided by Dr Angela Thompson from the CDC, Atlanta, USA; *tcd*A^+^ and *tcd*B^+^ (positive for the presence of toxins TcdA and TcdB); CDT: (+) positive or (-) negative for the presence of binary toxin genes; SH: system hypertension; DM: diabetes mellitus; EVA: haemorrhagic encephalic vascular accident; HIV+: positive for the human immunodeficiency virus.

*Laminin-1 adhesion assay* - Laminin-1 obtained from Engelbreth-Holm-Swarm mouse tumor EHS (LMN-1; Invitrogen), was immobilised on glass coverslips that had previously been placed into 24-well culture plates.^10^ To prepare the plates, 20 μg/mL of LMN-1 was suspended in 10 mM Tris-HCl, pH 6.6 and coated with glass coverslips for 18 h at room temperature (RT). To avoid non-specific association, coverslips were washed gently with 1x phosphate-buffered saline (PBS) containing 0.1% bovine serum albumin (BSA) (w/v), and then blocked with 1% BSA for 1h at RT. Before the bacterial inoculum, a final wash with 1x PBS containing 0.01% Tween 20 was performed.

All steps of the adhesion assay were carried out under anaerobic conditions. *C. difficile* strains were grown in BHI-PRAS overnight at 37ºC. All samples were adjusted to an OD_600_ of 0.4 (approximately 10^8^ CFU/mL) in an anaerobic buffer containing 1% BSA and 0.01% Tween 20 (0.1 g/L of magnesium sulfate; 0.2 g/L monobasic potassium phosphate; 3 g/L sodium chloride; 1.15 g/L dibasic sodium phosphate; 0.2 g/L of potassium chloride; 1 g/L sodium thioglycolate; 2 mL/L resazurin and 0.5 g/L cysteine). For the experiment, 300 μL of the sample were added to each well of the 24-well culture plates containing the LMN-1 coated coverslips and incubated for 1 h at 37ºC.^28^ After that, a final wash with 1x PBS containing 0.01% Tween 20 was performed, followed by 30 min for sample fixation with 3.7% formaldehyde. A second experiment was carried out under the same conditions, but with the bacterial strains grown in BHI-PRAS with an additional of 0.2%, 0.5%, or 1% glucose (Sigma-Aldrich) for 24 h, 48 h and 72 h to assess the effect of glucose on bacterial growth and adhesion to LMN-1. For the negative control, coverslips were incubated only with *C. difficile* strains and 2% of BSA (without LMN-1).

All coverslips were washed twice with distillated water before being incubated with 250 μL of Live/Dead^®^ BacLight™ Bacterial Viability Kit (Life Technologies) staining solution (3 µL of the dye mixture for each mL de distillated water). The plate was slowly agitated in the dark for 15 min under slow agitation. A fluorescence microscope (Carl Zeiss, Inc.) was used to capture the images.^28^ Bacterial adhesion was measured by counting an average of 20 random fields with the ImageJ software (Version 1.6.0_24) after. The assay was carried out in triplicate.

*Chemical treatments* - *Clostridioides difficile* RT012 was subjected to two different treatments in order to characterise the chemical nature of the molecule responsible for the adhesion, such as: incubation with proteinase K, a serine protease that hydrolyses a variety of peptide bonds (15 μg/mL), and with trypsin, which cleaves peptides at the C-terminal side of lysine and arginine amino acid residues (20 μg/mL). Both proteases were diluted in 0.1 M PBS pH 7.4 and incubated at 37ºC for 1 h. Alternatively, cells were also incubated for 1 h at 37ºC with 100 mM sodium periodate (glycoprotein oxidation) diluted in 0.05 M sodium acetate buffer pH 5.0.^10^ The bacterial inoculum was prepared using the same method as the LMN-1 adhesion assay.

*Scanning electron microscopy (SEM)* - SEM analysis was used to characterise the *C. difficile* matrix structure, as described in Pantaléon,^29^ with modifications. In brief, *C. difficile* strains were grown, according to the bacterial strains and culture conditions item, onto coverslips with a diameter of 9 mm (Knittel^®^) in order to analyse the biofilm production after 24 h. Samples were fixed in for 30 min with 2.5% glutaraldehyde in 0.1 M sodium cacodylate buffer and then post-fixed with for 15 min in 1% osmium tetroxide. Samples were dehydrated in series of ethanol concentrations (15%, 30%, 50%, 70%, 90% and 100%), critical point‐dried in CO_2_ and assembled on specimen stubs. Stubs were sputtered with a thin layer of gold using a Balzer’s apparatus and examined in a Quanta 250 scanning electron microscope (FEI Company), at Centro Nacional de Biologia Estrutural e Bioimagem (CENABIO) at UFRJ.

*Immunostaining for transmission electron microscopy* - Cells were grown in BHI-PRAS broth with 0.5% of glucose for 18 h before being washed twice with 1 mL of 0.1 M PBS and centrifuged for 5 min at 3000× g. Samples were adjusted to the McFarland standard tube 6 (~1.8 x 10^9^ CFU/mL) and bacterial cells were fixed in a solution containing 2% formaldehyde in 0.1 M PBS for 30 min at RT. After 30 min on formvar carbon-coated copper grids, the samples were blocked with 3% BSA in PBS for 30 min. Then, the grids were incubated with 20 µg/mL LMN-1 (Sigma-Aldrich) in Tris-HCl for 60 min at RT and washed with PBS twice and then incubated with a primary antibody mouse anti-laminin IgG (1:500; Sigma-Aldrich) in 1% BSA in PBS, for 60 min. After two PBS washes, the grids were incubated for 60 min with a secondary antibody anti-mouse IgG conjugated to colloidal gold particles (1:50; Au 10 nm - TED PELLA, INC.). The cells were washed twice with 3% BSA, once in 1% BSA, and once with in PBS. After 5 min of fixation in 1% glutaraldehyde in PBS, the grids stained for 30 min with 5% uranyl acetate. The investigation was carried out with a Zeiss 900 transmission electron microscope.

*Whole proteins extract* - To obtain protein extracts of *C. difficile* RT012, an inoculum from 18 h growth was made in 4 mL of BHI-PRAS broth containing 0.5% glucose, for 24 h at 37ºC in anaerobiosis. Subsequently, the growth broth of RT012 of *C. difficile* was centrifuged at 2700x g for 15 min at RT, the supernatant was discarded and the sediment washed 1x with sterile PBS (0.1M; pH 7.2) and, centrifuged again under the same conditions. The material was mixed with 10 mL of ice-cold acetone (analytical grade), allowed to stand on ice for 5 min and then centrifuged at 7000x g. The SpeedVac (Savant™ SPD131DDA SpeedVac™ Concentrator; Thermo Scientific) was used to remove residual acetone, and proteins were obtained by dissolving the dry extract with 0.2 mL of 1% sodium dodecyl sulfate (SDS) (Bio-Rad).^30^ The protein extracts were kept at -20ºC, until the moment of use. Protein quantifications were determined using the Qubit Protein assay kit (Life Technologies) according to the manufacturer’s instructions.

*Affinity chromatography column* - The affinity column was prepared by mixing 0.5 mL of Affi-gel^®^10 (Bio-Rad) with a solution of 1 mg/mL LMN-1 in 0.1 M PBS, pH 7.2. After 24 h incubation at 4ºC, 50 mM ethanolamine pH 7.8 was added to the column and incubated for 1 h. The column was washed twice with 0.1 M PBS, pH 7.2, followed by 2 M NaCl at pH 7.0. Proteins were passed through the column three times. To remove any unbound proteins, the column was then washed with 0.1 M PBS. Proteins were then eluted from the column with NaCl at concentrations of 0.25, 0.5, 1.0 and 2.0 M. After that, the eluted proteins were collected and desalted using a Centricon (Amicons^®^ - Sigma-Aldrich).^31^

*Protein identification by mass spectrometry* - The eluted proteins were analysed by sodium dodecyl sulphate-polyacrylamide gel electrophoresis (SDS-PAGE) method (1D gel) and slices of gel containing these proteins were placed in 1.5 mL sterile tubes. Then, all steps were performed in a laminar flow, and all solutions were prepared using HPLC water (TEDIA^®^). Two hundred microliters of Ammonium Bicarbonate (AMBIC - 25 mM ammonium bicarbonate buffer, pH 8.0 with 1 mM calcium carbonate) in 50% acetonitrile (ACN) (TEDIA^®^) were added to the tubes and incubated for 15 min at RT. This process was repeated three times. Thereafter, the gel slices were washed with ACN for 5 min before drying in SpeedVac (Savant™ SPD131DDA SpeedVac™ Concentrator; Thermo Scientific) for 30 min. After drying, 30 µL of 10 mM dithiothreitol (DTT) solution (Sigma-Aldrich) in 25 mM AMBIC was added and the tubes were incubated at 56ºC for 1 h. After removing the DTT solution, 30 µL of 55 mM iodoacetamide (Sigma-Aldrich) in AMBIC (fresh prepared) were added to the tubes and incubated at RT for 45 min in the dark. The iodoacetamide solution was discarded, and the gel slices were washed twice with 400 µL of 25 mM AMBIC for 10 min. And, afterwards, the gel pieces were soaked in 200 µL ACN for 5 min before drying in a SpeedVac for 30 min.^32^ After that, the gel slices were enzymatic digested by rehydrating them with 13 µL of the trypsin solution (20 µg/mL in AMBIC) per gel band, followed by an overnight incubation at 37ºC.^33^ Following this, 30 µL of a solution containing 5% formic acid and 50% ACN was added and incubated for 1 h at RT. This solution was transferred to new tubes, and the procedure was repeated two times. Subsequently, the samples were dried in a SpeedVac and pellets were suspended in 10 µL of 0.1% formic acid. The tubes were sealed and kept at -80ºC to be analysed in a Nano-HPLC-MS/MS (Orbitrap) mass spectrometer at proteomics platform of Fundação Oswaldo Cruz - Fiocruz.

*Bioinformatics analysis* - The peptides analysed in the mass spectrometer generated mass spectra in tandem, which were analysed by Peaks program (Bioinformatics Solutions Inc.), using the NCBInr database. Thereafter, the identified proteins were analysed in PsortB program v. 3.0 to predict the subcellular location of identified proteins,^34^ BLAST tool (Basic Local Alignment Search Tool) for genetic identity comparisons (https://blast.ncbi.nlm.nih.gov/Blast.cgi), SignalP for prediction of the presence and location of signal peptide and (UniProtKB) was used for molecular, cellular and biological function of proteins.^35^

*Statistics analysis* - The statistical analysis was performed using the GraphPad Prism^®^ software (Version 5.01, GraphPad Software, La Jolla, CA, USA). The Student’s t-test (unpaired) was used for comparisons. The statistically significant differences were indicated using p-value.

## RESULTS

**Fig. 1:**
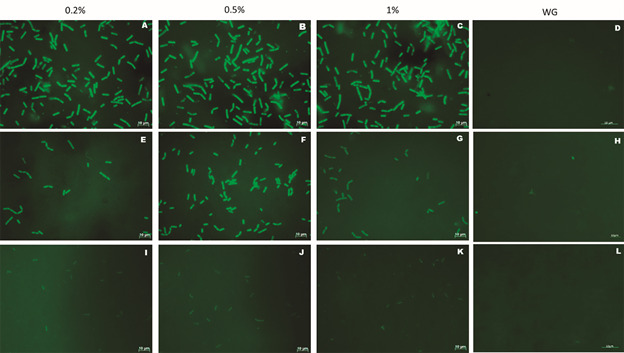
adherence test of *Clostridioides difficile* ribotypes (24 h growth) to laminin-1 by fluorescence microscopy using different concentrations of glucose (0.2%, 0.5% and 1%) and live/dead staining. Green spots represent active, membrane-intact bacteria. A, B, C and D correspond to the adherence assay of RT012 (CD630 strain); E, F, G and H the adherence assay of RT027 (BI/NAP1) and I, J, K and L the assay of RT135 (SJ1 strain). WG: without supplementation of glucose.

**Fig. 2:**
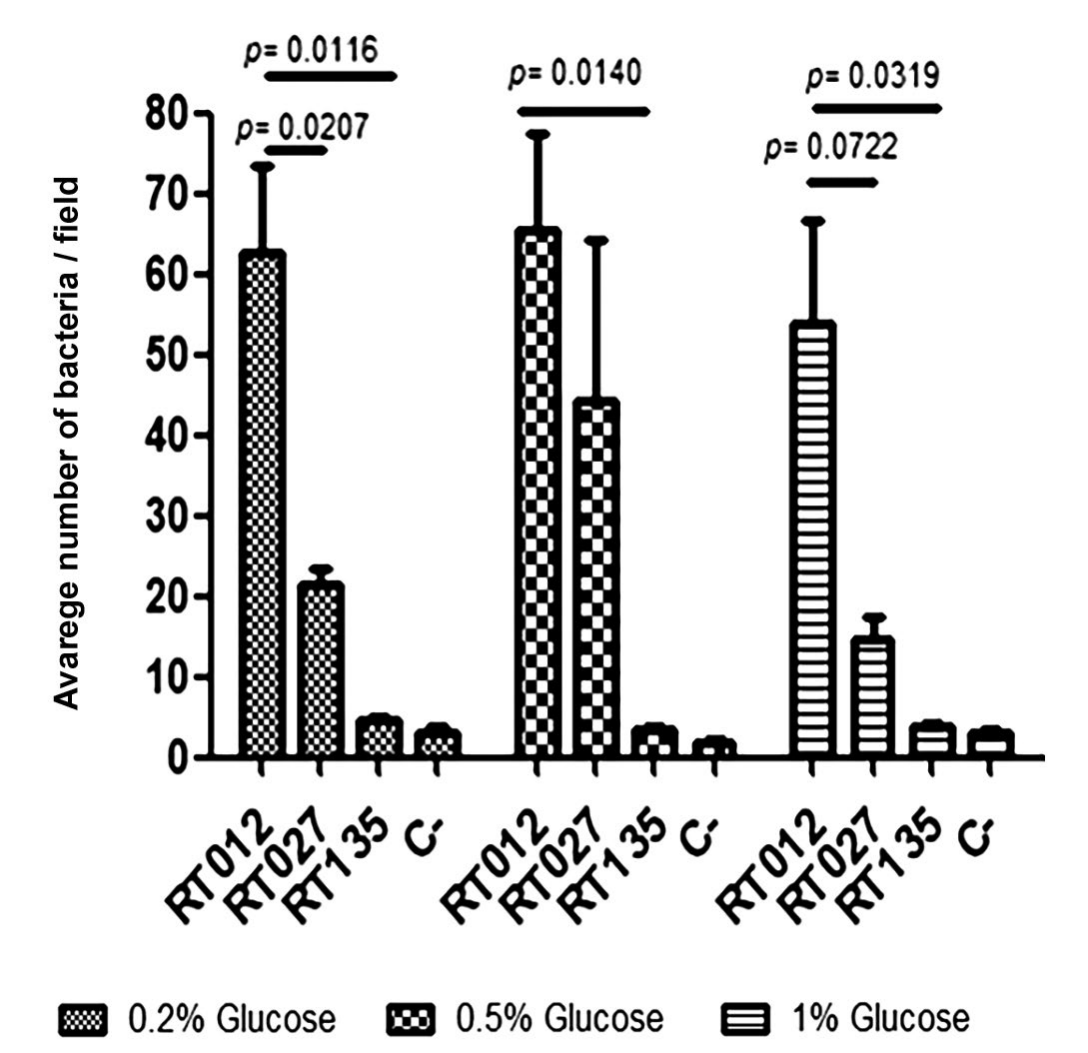
quantitative adhesion assay of *Clostridioides difficile* ribotypes (24 h growth) to laminin-1 at different glucose concentrations (0.2%, 0.5% and 1%). All tests were performed in triplicate and the bars of the standard deviation corresponded to the mean of the three technical replicates. Results were considered significant when p < 0.05 (Student’s t unpaired test). Bovine serum albumin (BSA) 2% was used as a negative control (C-).

*Binding of C. difficile strains to LMN-1* - After 24 h of growth, fluorescence microscopy was used to assess the adherence of *C. difficile* ribotypes (RT012, RT027, RT135, RT014 and RT133) to LMN-1. First, all ribotypes were grown without glucose and adhesion assay to LMN-1 was performed. However, none of the ribotypes showed any signs of adherence. Regardless, after cultivating them in the presence of glucose at three different concentrations (0.2%, 0.5% and 1%), RT135, RT012 and RT027 were able to attach to LMN-1 (Fig. 1). Ribotypes 014 and 133 did not adhere to LMN-1 (data not shown) at any of the glucose concentrations tested. To quantify their adherence to LMN-1, all ribotypes were grown with three different concentrations of glucose, 0.2%, 0.5% and 1%. *C. difficile* ribotypes RT012 and RT027 adhered to LMN-1 more strongly than RT135 (Fig. 2).

**Fig. 3:**
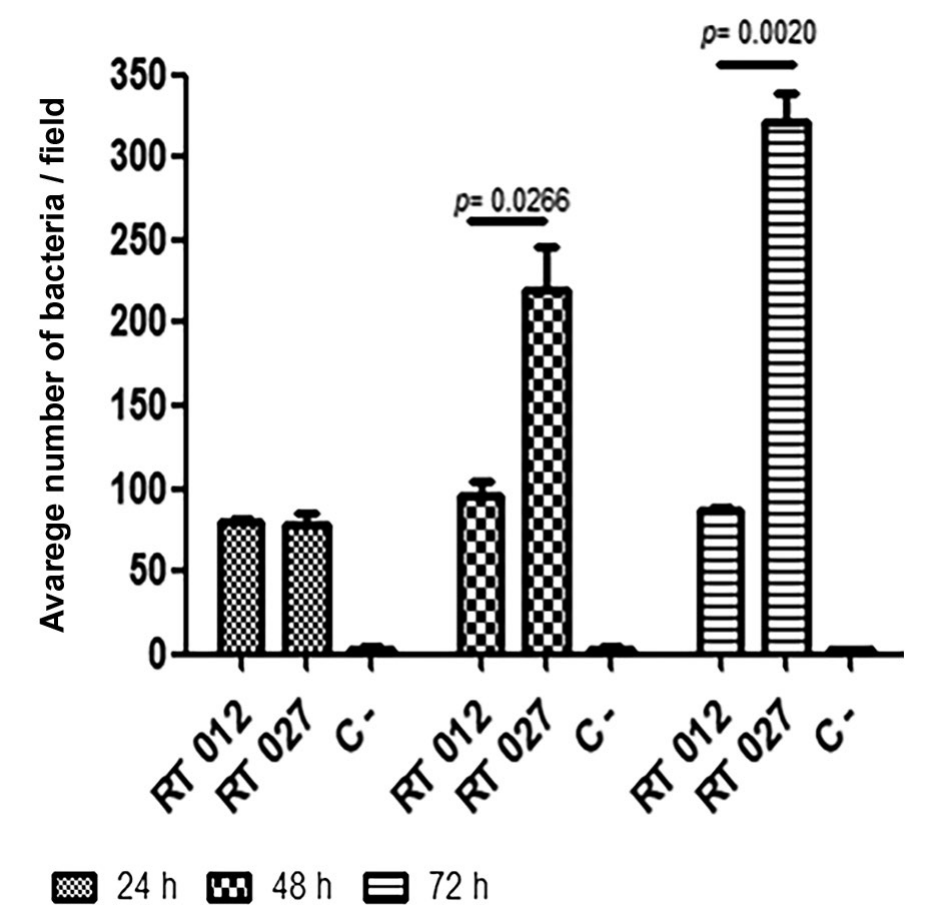
adhesion test of ribotype RT012 (CD630) and RT027 of *Clostridioides difficile* strains to laminin-1 with 0.5% glucose and at different growth times (24 h, 48 h and 72 h). Bovine serum albumin (BSA) 2% was used as negative control (C-), without LMN-1. Results were considered significant when *p* < 0.05.

*Quantifying the binding of C. difficile strains to LMN-1* - After determining the optimal glucose concentration for maximum *C. difficile* adhesion to LMN-1, an adhesion assay was performed to evaluate influence of the incubation period (Fig. 3). Ribotypes 012 and 027 were chosen for their high adherence to LMN-1. Incubation period had no effect (p = 0.1535) on RT012 strain adhesion capacity (Fig. 3). RT027, on the other hand, showed increased adherence (p = 0.0055) only after 48 and 72 h of culture (Fig. 3).

**Fig. 4:**
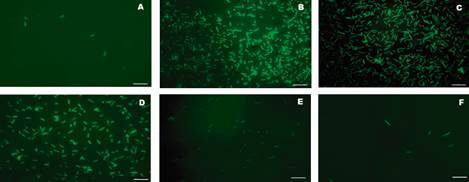
inhibition assay ribotype 012 (CD630) of *Clostridioides difficile* strains after chemical and enzymatic treatments. A - Proteinase K (15 µg/mL); B - Trypsin (20 µg/mL); C - sodium periodate (MPS; 100 mM); D - Bacteria without treatment; E - proteinase K (15 µg/mL) follow by MPS (100 mM); F - Negative control Bovine serum albumin (BSA) 2%, without LMN-1. All experiments were performed in triplicate. Magnification: 1000x.

**Fig. 5:**
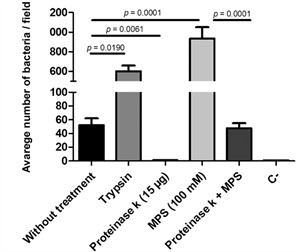
quantification of the adhesion assay of *Clostridioides difficile* RT012 (CD630) against laminin-1 after chemical and enzymatic treatment. As negative control (C-) Bovine serum albumin (BSA - 2%) inoculated with strain CD630, without laminin, was used. The bars represent the standard deviations (*) of the means of two technical and two biological experiments. MPS: sodium metaperiodate. The results were considered significant when p < 0.05.

The molecular nature of the bacterial surface molecule involved in *C. difficile* binding to LMN-1 was investigated. The proteinase K treatment prior to the adhesion assay resulted in a significant reduction in the bacteria’s binding rates, as shown in Fig. 4. When the samples were treated with trypsin and sodium metaperiodate (MPS) the binding rates to LMN-1 increased. When compared to the untreated sample, treatments with proteinase K (p = 0.0061), trypsin (p = 0.0190) and MPS (p = 0.0001) affected binding (Fig. 5). When cells were first treated with proteinase K and then with MPS, adhesion to LMN-1 reduced compared to treatment with MPS alone (p = 0.0001). There was no increase in LMN-1 adhesion when only proteinase K was used.

**Fig. 6:**
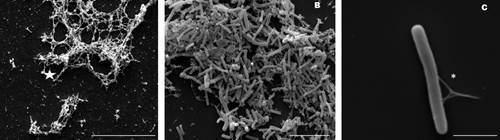
scanning electron microscopy (SEM) showing *Clostridioides difficile* RT012 (CD630) on glass coverslips at 24 h with 0.5% glucose. A - micrograph showing an extracellular fibrous-biofilm matrix (star) produced by *C. difficile*; B - micrograph showing *C. difficile* aggregation by the biofilm; C - Detail of the biofilm produced by *C. difficile* connecting the cell to the glass support (*). Scale bars: A - 20 μm; B - 10 μm; C - 2 μm.

**Fig. 7:**
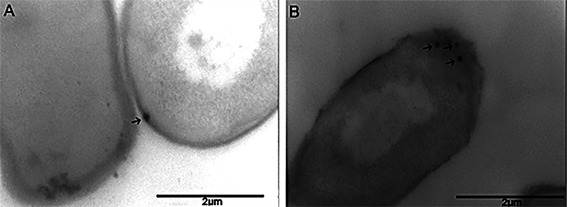
immunoelectron microscopy of RT012 (CD630) from *Clostridioides difficile*. Cells with 24 h growth with 0.5% of glucose were incubated with laminin-1. Micrographs A and B show *C. difficile* cells incubated with LMN-1, treated with the anti-LMN-1 antibody and anti-IgG conjugated to colloidal gold beads. Arrows indicate recognition on the surface of bacterial cells. Scale bar: 2 μm.

*Matrix polymer production* - SEM was used to examine growth on glass coverslips for *C. difficile* RT012 (Fig. 6). An extracellular fibrous-biofilm matrix was observed after 24 h of culture (Fig. 6A). *C. difficile* attachment to the glass coverslips was facilitated by the matrix (Fig. 6C). Furthermore, the intertwining biofilm appeared to aid bacterial aggregation (Fig. 6B).

*Immunoelectron microscopy of the interaction C. difficile Ribotype 012 and LMN-1* - An immunolabeling assay with colloidal gold particles was used to confirm the interaction *C. difficile* adhesin and LMN-1. Fig. 7 depicts positive labeling on the bacterial surface following interaction with LMN-1. The labeling was slight and visible in some cells. This finding suggests that this adhesin is present and exposed on the surface of *C. difficil*e.

*Protein identification by mass spectrometry* - The total protein extract was passed through an affinity chromatography column, and the elute applied to an SDS-PAGE to identify the protein(s) responsible for the recognition of LMN-1 by the RT012 *C. difficile*. Mass spectrometry was used to analyse all proteins present in the gel, and 14 proteins were identified (Supplementary data - Table). All proteins recognised were evaluated in the PsortB program to predict the cell localisation, revealing that four proteins (WP_021421450.1, WP_022618559.1, WP_021380039.1 and WP_021367719.1) were in the membrane. When UniProtKB database and SignalP were used to analyse the biological function of proteins, only one protein was identified with an adhesion function, a signal peptide (99% of chance - SEC/SPI secretion system) and with an extramembranous portion, the S-layer protein, SlpA (Accession numbers: WP_022618559.1).

## DISCUSSION

Colonisation requires adhesion to host tissue cells, which is the first critical stage in most bacterial infections.^36^ MSCRAMMs are bacterial adhesins that can recognise ECM (proteoglycans, glycoproteins and fibrous proteins) on the cell host and can help them colonise and spread.^13^ ^,^ ^37^ Laminin is the main component of the ECM and inflammatory processes and epithelial damages can expose patches of laminin or make it thicker in the basal lamina, providing a constant and ubiquitous availability of ligands for bacterial laminin surface receptors.^38^ ^,^ ^39^

The ability of multiple *C. difficile* ribotypes to interact with LMN-1 was investigated, in this study, including the autochthonous (exclusive) Brazilian strains RT133 and RT135. The adhesion test was initially performed using a BHI-PRAS broth culture medium, but no adhesion was observed. Kreutz and Jürgens^36^ tested the ability of 70 *Clostridium* spp. strains, including *C. difficile* (n = 24), to identify fibronectin and laminin. The bacteria were cultured in Rosenow broth, and the experiment used latex particles coated with ECM components. Rosenow broth composition is like that of BHI broth that contains 0.2% glucose. Only three of the 24 *C. difficile* strains studied were found to identify LMN-1 weakly, and incubating laminin-coated latex beads with antibody anti-LMN greatly reduced the ability of *C. difficile* to recognise LMN-1. When the authors used the Schaedler agar and decided to repeat the agglutination assay with LMN-1 and *C. difficile*, they observed an increase in the recognition for seven out of 24 strains, of which five were weakly adherent and two substantially adherent.^36^ In conclusion, Schaedler agar increased all strains’ binding to fibronectin and LMN, and the adhesion of *C. difficile* to LMN was stronger. According to the authors, the media may have also increased the formation of *C. difficile* adhesins that recognise LMN. Like BHI, Schaedler agar is used to cultivate a variety of anaerobic bacteria species; however, it contains 5.8 g/L glucose. Based on this, all *C. difficile* ribotypes were tested for adherence to LMN-1 again by cultivating in BHI-PRAS varying amounts of glucose (0.2%, 0.5% and 1%). Thus, in the presence of 0.2% glucose, binding to LMN-1 by ribotypes 027, 012 and 135 was observed within 24 h. Nonetheless, when 0.5% glucose was added to the medium, RT012 and RT027 adhesion increased significantly, but not for RT135. Furthermore, when 1% glucose was added, only for RT027 and RT135 showed a substantial decrease. As a result, when bacteria from ribotypes 012 and 027 were incubated with 0.5% glucose, they had the highest adhesion capacity. In addition, a growth curve for RT012 was performed in the absence and presence different glucose concentrations supplementation. When there was no glucose in the culture medium, the *C. difficile* growth curve reached the death stage earlier than the other growth curves that had the glucose supplementation (data not shown). Then, using 0.5% glucose**,** different growth times (24 h, 48 h and 72 h) were examined, and RT027 was found to increase over time. However, the increase of glucose concentration or growing time had no effect on RT012 adhesion.

*Clostridioides difficile* has been shown to be able to metabolise a wide range of carbohydrates, including fructose, sucrose and glucose.^40^ Glucose is the most abundant energy source for cell biosynthesis and some metabolic processes. This monosaccharide can stimulate the survival and propagation of various pathogenic bacteria in low nutrient environments.^41^ ^,^ ^42^ It has been previously demonstrated that adhesin expression can be influenced by a variety of environmental conditions, and that it is more prevalent when specific nutrients, such as glucose, are present. This emphasises the importance of an energy source in the adhesion process, as Antunes et al.^43^ reported that approximately 18% of all *C. difficile* genes, with catabolite control protein A (CcpA) regulating 50% of these genes.^43^ The genes regulated a variety of activities, including the utilisation of specific carbon sources, distinct fermentation pathways, amino acid metabolism and toxin production in response to glucose availability. When *C. difficile* grown in the presence of 0.5% glucose, it has been shown to use several carbohydrates that may be important during the pathogenesis by promoting survival and growth in the intestine.^43^ The authors also demonstrated that the presence of fast carbohydrate metabolisation, such as glucose, suppressed transcription of *tcd*A and *tcd*B.^43^ Following that, simple dietary sugars (glucose or fructose) were shown to improve host colonisation and spore-mediated *C. difficile* transmission.^44^ In contrast, Rungrassamee et al.^45^ demonstrated in *Escherichia coli* that oxidative stress induces glucose transport in a glucose phosphotransferase system (*ptsG*)-dependent manner. The gene was involved in the early stages of glucose metabolism. As a result, the fact that RT027 is an epidemic strain, it is a high toxin and spores producer, and has a higher adherence after 48 h suggests that oxidative stress may be involved in this process.

*Clostridioides difficile* is heterotrophic, saccharolytic, proteolytic bacterium with multiple on amino acids and sugar-based energy production pathways.^40^ ^,^ ^46^ According to Theriot and Young, metabolomic studies in the gastrointestinal tract detected many of the nutrients that support *C. difficile* growth and toxin production, such as bile acids, carbohydrates and amino acids.^40^ Thus, during dysbiosis, there is a shift in the predominant members of the gut microbiota, which may alter bacterial metabolism in the gut and allow *C. difficile* colonisation.^40^ Furthermore, despite the fact that only a few studies have been conducted, it has been reported that *C. difficile* is capable of utilising carbon sources such as succinate or simple sugars like glucose and fructose for optimal growth in the gastrointestinal tract.^47^ ^,^ ^48^ Other studies have discovered adhesins on the surface *C. difficile* that are synthesised in the absence of glucose in the medium.^19^ ^,^ ^21^ ^,^ ^49^ Biofilm may have played a role in this recognition because the adhesion occurred only in the presence of glucose. Even though the SEM images revealed an immature polymer matrix rather than a mature biofilm, it implies that, while glucose influences RT012 adherence to LMN-1, the biofilm appears to be uninvolved in this recognition. It is worth noting that biofilm assays for *C. difficile* strains are typically performed with BHI-PRAS containing 1.8% glucose and are evaluated after 72 h.^29^ ^,^ ^50^ Given that the assay was performed in same media, but with 0.5% glucose, and there was insufficient time for the biofilm to form after 24 h, it appears that another surface adhesin is involved in the LMN-1 recognition.

The ability of pathogens to recognise the ECM have been studied in several bacterial species.^14^ ^,^ ^51^ Pathogenicity of *Staphylococcus* spp., which can cause a variety of life-threatening infections, is dependent on direct binding to ECM and/or host cells. There are at least 20 adhesins involved in Staphylococcal mechanisms of adherence to and internalisation into host cells regarding of MSCRAMMs.^52^ For the genus *Yersinia* adherence to laminin may contribute to tissue invasion and blood dissemination.^53^ *Yersinia enterocolitica* and *Y. pseudotuberculosis*, both enteropathogenic bacteria, can cause several diseases, including intestinal and extraintestinal disorders. Several pathways for adhesion to eukaryotic cell membranes have previously been identified, including YadA, an outer membrane protein, YadA that mediates specific attachment of *Y. enterocolitica* and *Y. pseudotuberculosis* to laminin, collagen, and fibronectin. YadA inhibits serum complement activation, which is required for virulence.^54^ *Bacillus anthracis*, the anthrax etiological agent, has also been found to express BslA, an S-layer protein involved in mammalian infection. By recognising LMN, BslA increases the vegetative form of the bacteria’s attachment to host cells. The interaction allows infection in organs and penetration through the blood-brain barrier in vivo.^55^

To assess *C. difficile* adhesion to LMN-1 and to perform additional analysis the RT012^19^ ^,^ ^21^ ^,^ ^49^ was chosen. Proteinase K, sodium periodate, and trypsin were used to try to identify the chemical nature of the adhesin that recognises LMN-1 in RT012 cells. Proteinase K treatment reduced RT012 adhesion to LMN-1, whereas trypsin increased adhesion. This could be due to peptides cleavage at various adhesion-promoting sites. Trypsin cleaves peptides at the C-terminal side of lysine and arginine residues, whereas proteinase K preferentially cleaves peptide bonds adjacent to the carboxyl group of aliphatic and aromatic amino acids. Furthermore, when the lysine and arginine residues are adjacent to the amino acids in sequence to the peptides on the C-terminal side of the lysine and arginine residues, the cleavage rate is slower. As a result, perhaps the trypsin test should be performed longer than 1 h to see if the same effect as seen with proteinase K could be observed. Regardless, the *C. difficile* adhesin is most likely to be protein, as reduced adhesion to LMN-1 with proteinase K was observed. In contrast, sodium periodate increased RT012 adhesion to LMN-1. When cells were treated with proteinase K first, then with MPS, the adhesion to LMN-1 was drastically reduced compared to the 100 mM MPS treatment and equaled that of untreated cells. Because MPS acts by oxidising hydroxyl groups on adjacent carbon atoms in sugar moieties, the structure of glycoproteins is disrupted, releasing sugar residues that can form a cross link between LMN-1 and a cell.^56^ ^,^ ^57^ ^,^ ^58^ Our findings suggest that the adhesin that recognises LMN-1 is most likely a glycoprotein.

Because most *C. difficile* adhesins have been described as being on the bacterial surface,^59^ immunoelectron microscopy was used to determine whether the cellular localisation of the adhesin responsible for recognition LMN-1 was correct. Despite the weak labeling of the protein, the molecule was still found on the surface and present in the majority visualised cells, indicating that the protein is poorly exposed. This finding is explained by Robinson et al.^60^ explain this finding by demonstrating that the size of colloidal gold particles can influence the immunostaining efficiency or make them difficult to detect using electron microscopy. TEM, on the other hand, confirmed that the adhesin is stochastically located on the bacterial surface. Adhesion is a complex process and the ability of microorganisms ability to express a wide range of adhesins on their surface may explain the adaptability of both microbiota and opportunistic pathogens.^10^ Despite the fact that the exposure of this adhesin on the surface of *C. difficile* appeared to be low, our experiments show that the molecule is constantly induced and can be expressed under a certain environmental conditions. As a result, different cultivation times, temperatures, and pH variations in the culture medium should be studied.

Following that, 14 proteins from the total extract were identified using mass spectrometry, but only one of them, SlpA, had an adhesion function and was located on the cell surface. This protein is found in *C. difficile* S-layer (SLPs). SLPs are cell wall-anchored proteins that highly glycosylated in some organisms.^61^ SlpA is abundantly present on the cell surface of *C. difficile* and accounting for 15% of the cell’s total protein.^62^ ^,^ ^63^ SlpA is synthetised as a preprotein, which is then secreted and cleaved into the LMW and HMW subunits of SLP by Cwp84. SLPs have already been described in *C. difficile* as providing structural integrity to the cells, acting as molecular sieves, binding to host tissues and extracellular matrix proteins.^64^ ^,^ ^65^ Both SLP subunits have been shown to be capable of adhering *in vitro* to human gastrointestinal tissue and intestinal epithelial cells.^65^ ^,^ ^66^ Thus, SlpA plays an important role in *C. difficile* infection, and it may be an appealing target for intervening in the intestinal colonisation process by this pathogen.^66^

Despite the intriguing data obtained in this study, additional *in vitro* analyses are required to demonstrate that SlpA interacts with LMN-1. Nonetheless, this is the first study to identify and characterise an adhesin in *C. difficile* capable of recognising this component of the extracellular matrix, LMN-1. Future strategies will center on identifying the binding molecule, which may help us understand *C. difficile* colonisation in the colon. It may also lead to the identification of potential protein targets as well as the understanding of *C. difficile* colonisation and inflammation.
