# Supplementary material for: Binding of the extracellular matrix laminin-1 to Clostridioides difficile strains
Source: Mem Inst Oswaldo Cruz. 2022 Jun 17;117:e220035. doi: 10.1590/0074-02760220035 (PMC9208321; doi:10.1590/0074-02760220035)
Supplement: Supplementary file 1 [file 1678-8060-mioc-117-e220035-s.pdf]

TABLE

Identification of total and flagellar proteins of RT012 (CD630 strain) of the *Clostridioides difficile* that recognised Laminin (LMN) by the affinity column

Whole protein	Access number	Molecular mass	Size	gene	Location	Function
penicillin-binding transpeptidase [Clostridioides difficile]	WP_021383830.1	111336	992 aa	-	Transmembrane	Adherence to penicillin
regulator of chromosome condensation (RCC1) repeat family protein [Clostridioides difficile]	WP_021382676.1	167264	1522 aa	-	cytoplasm	inhibitor of β-lactamase
acetyl-CoA acetyltransferase [Clostridioides difficile]	WP_021421450.1	40846	391 aa	*thlA*	Membrane	Activity catabolic[a]
repeat protein of tetratricopeptide	WP_021419633.1	59680	501 aa	-	-	-
DNA/RNA helicase [Peptoclostridium difficile 630]	WP_011860792.1	336307	2907 aa	-	Cytoplasm	Helicase
reactivating factor for ethanolamine ammonia lyase [Clostridium difficile 630]	WP_022620214.1	52400	477 aa	*eutA*	Cytoplasm	Lyase
S-layer protein [Clostridioides difficile]	WP_022618559.1	64703	610 aa	*slpA*	Membrane	Adhesion protein
ADP-ribosyltransferase exoenzyme family protein [Clostridioides difficile]	WP_021372011.1	64755	472 aa	-	Cytoplasm	-
protein containing C- terminal domain de 5'- nucleotidase	WP_021380039.1	64778	601 aa	-	Membrane	Nucleotidase
gamma-glutamyltransferase [Clostridioides difficile]	WP_021384296.1	59818	537 aa	ggt	Cytoplasm	Glutathione; hydrolase
Imidazole-4-carboxamideisomerase	WP_003429789.1	26681	240 aa	*hisA*	Cytoplasm	Activity catabolic[b]
HydroxyisocaproylCoA	WP_003427763.1	42365	375 aa	*hadC*	Transmembrane	Cofactor
protein containing domain DUF1700	WP_021367719.1	40646	352 aa	-	Membrane	-
Integrase Tn916-like, CTn1-Orf1 of type tyrosine	WP_021384652.1	45648	393 aa	*int*	Transmembrane	Integrase
Flagellar proteins	Access number	Molecular mass	Size	gene	Location	Function
S-layer protein [Clostridioides difficile]	WP_021372165.1	75346	713 aa	*slpA*	Membrane	Adhesion protein
Protein from the FtsX-like family of permeases	WP_021415351.1	100027	886 aa	-	Membrane	-

a: this protein is involved in step 1 of the sub-pathway that synthesises (R)-mevalonate from acetyl-CoA; *b*: this protein is involved in step 4 of the sub-pathway that synthesizes L-histidine from 5-phospho-alpha-Dribose 1-diphosphate.

CC BY
